# Chemiluminescence in Combination with Organic Photosensitizers: Beyond the Light Penetration Depth Limit of Photodynamic Therapy

**DOI:** 10.3390/ijms232012556

**Published:** 2022-10-19

**Authors:** Jie Gao, Zhengjun Chen, Xinmin Li, Mingyan Yang, Jiajia Lv, Hongyu Li, Zeli Yuan

**Affiliations:** 1 Key Laboratory of Basic Pharmacology of Ministry of Education and Joint International Research Laboratory of Ethnomedicine of Ministry of Education, Zunyi Medical University, No.6 West Xuefu Road, Xinpu District, Zunyi 563000, China; 2 Key Laboratory of Biocatalysis & Chiral Drug Synthesis of Guizhou Province, School of Pharmacy, Zunyi Medical University, No.6 West Xuefu Road, Xinpu District, Zunyi 563000, China; 3 Guizhou International Scientific and Technological Cooperation Base for Medical Photo-Theranostics Technology and Innovative Drug Development, Zunyi Medical University, No.6 West Xuefu Road, Xinpu District, Zunyi 563000, China

**Keywords:** chemiluminescence, photodynamic therapy, photosensitizer, chemiluminescence resonance energy transfer, reactive oxygen species

## Abstract

Photodynamic therapy (PDT) is a promising noninvasive medical technology that has been approved for the treatment of a variety of diseases, including bacterial and fungal infections, skin diseases, and several types of cancer. In recent decades, many photosensitizers have been developed and applied in PDT. However, PDT is still limited by light penetration depth, although many near-infrared photosensitizers have emerged. The chemiluminescence-mediated PDT (CL-PDT) system has recently received attention because it does not require an external light source to achieve targeted PDT. This review focuses on the rational design of organic CL-PDT systems. Specifically, PDT types, light wavelength, the chemiluminescence concept and principle, and the design of CL-PDT systems are introduced. Furthermore, chemiluminescent fraction examples, strategies for combining chemiluminescence with PDT, and current cellular and animal applications are highlighted. Finally, the current challenges and possible solutions to CL-PDT systems are discussed.

## 1. Introduction

Photodynamic therapy (PDT) has emerged as a promising and clinically valuable noninvasive treatment modality owing to its high spatiotemporal selectivity and low toxic side effects [1,2,3,4]. In recent decades, many photosensitizers (PSs) and related materials have been extensively developed and successfully applied in research on PDT [5,6,7,8,9,10,11,12]. Typically, PSs are a vital component of PDT. When a specific light wavelength is irradiated to PSs, they interact with molecular oxygen (^3^O_2_) to generate reactive oxygen species (ROS), which cause damage to their surrounding living materials (including lipids, nucleic acids, cell membranes, organelles, and proteins), thereby affecting normal cell function and thus inducing cell death [4]. PDT has been applied in dermatology, dentistry, and ophthalmology. In particular, PDT is widely used in cancer treatment and related research.

However, because of the limited penetration ability of visible light (<2 mm) and near-infrared (NIR) light (~1 cm) [13], clinical PDT is often used only for the treatment of superficial diseases, such as skin cancer. Therefore, various attempts and research have been conducted to overcome the limitation of light penetration depth, such as two-photon PSs, second NIR PSs, ultrasound, or X-rays instead of light to initiate PDT [14]. The chemiluminescence-mediated PDT (CL-PDT) is an external light-source-free strategy, and thus it can be theoretically applied to treat disease at any site and depth. Compared to conventional PDT, CL-PDT is particularly suitable for treatment of diseases in deep tissues and organs, such as liver cancer, brain tumors, bone cancer, etc.

Considering the widespread interest in PDT, many outstanding reviews have summarized the design of PSs, PDT applications, and outlooks [4,10,14,15]. However, a limited number of reviews sporadically mention the chemiluminescence integration of PDT without providing any comprehensive summary [16,17,18,19,20,21]. Instead, this review focuses on the rational introduction of chemiluminescence into PDT to achieve adequate therapeutic results. In addition, only organic systems are summarized. Specifically, PDT types, the vital role of excitation light in PDT, chemiluminescence concept and classification, common chemiluminescent molecules, and design and bio-application of CL-PDT systems are introduced. Furthermore, the selection of chemiluminescent fraction, strategies for combining chemiluminescence with PDT, and current cellular and animal applications are highlighted. Finally, possible optimal solutions for the CL-PDT system are suggested for future research considerations, and current issues requiring urgent attention are discussed.

## 2. PDT Types

**Figure 1:**
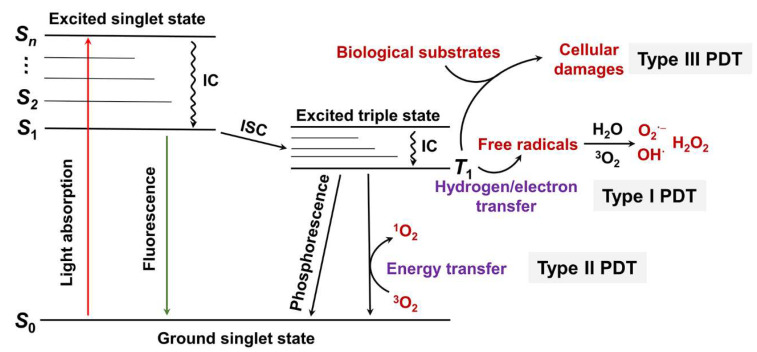
Jablonski’s diagram for the principle of type I, type II, and type III PDT. S_0_: the singlet ground state; S_1_: the first excited singlet state; S_2_: the second excited singlet state; S_n_: the n^th^ excited singlet state; IC: internal conversion; ISC: intersystem crossing.

In general, PDT utilizes oxygen for subsequent ROS production, and the amount of oxygen often affects PDT effectiveness. According to the pathway of ROS production by PSs, PDT is generally divided into type I and type II (Figure 1) [14]. When light with proper wavelength irradiates a PS (light absorbance), the PS is excited from the ground singlet state (S_0_ state) to the second excited singlet state (S_2_ state) or other excited singlet states (S_n_ state). The S_n_ state rapidly relaxes back to the first excited singlet state (S_1_ state) through an internal conversion. Then, the S_1_ state relaxes back to the S_0_ state through radiative (fluorescence) and non-radiative transitions. Additionally, the S_1_ state can convert to the triplet excited state (T_1_ state) via an intersystem crossing. The photon emission from the T_1_ state to S_0_ state is known as phosphorescence. In type I PDT, the triplet excited state of PS interacts directly with surrounding substrates to generate free radicals via electron transfer. Subsequently, triplet oxygen (^3^O_2_) and H_2_O react with the free radical anions to produce hydroxyl radical and superoxide anion, respectively. In type II PDT, the T1 state of PS converts the surrounding triplet oxygen molecules to highly reactive singlet oxygen via an energy transfer pathway. From the above, it is clear that type II PDT is more dependent on oxygen concentration. In contrast, type I PDT requires less oxygen and can work well under hypoxia conditions. Of course, both type I and type II mechanisms require oxygen to participate in the photochemical reaction process. However, type I PSs can reduce their dependence on oxygen by partially cycling oxygen. Sibata et al. proposed a type III PDT (Figure 1), completely different from the two mechanisms described above. The excited state PS interacts directly with biologically active molecules such as proteins, nucleic acids, and lipids, disrupting their function [22]. Type III PDT does not need oxygen, so it can work in hypoxic or even oxygen-free conditions but requires the PS to have specific targeting [23]. 

## 3. The Excitation Light in PDT

**Figure 2:**
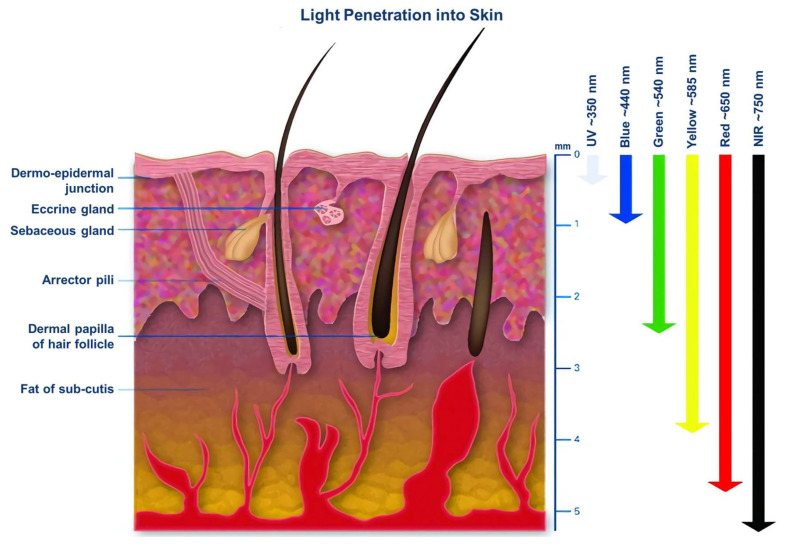
The tissue penetration depth of different light wavelengths [24]. Reprinted with permission from Ref [24] Copyright 2005 Taylor and Francis, Abingdon, UK.

In addition to the PSs, the excitation light wavelength is another essential factor for PDT. Different light wavelengths penetrate tissues at different depths owing to the absorption and scattering of light by tissues and biological components (Figure 2) [24]. For example, it was found that the penetration depth of long-wavelength NIR light (700–1100 nm) exhibited greater tissue penetration than that of short-wavelength ultraviolet–visible light (400–700 nm) by more than a twofold margin. This is because NIR light lies inside the so-called “optically transparent window” of biological tissues, giving it deeper penetration and less attenuation during tissue propagation [14]. However, the effect of NIR light on PDT in deeper tissues remains limited. Therefore, how to reach beyond the light source and thus achieve deeper PDT has become a new research area.

## 4. Chemiluminescence

**Figure 3:**
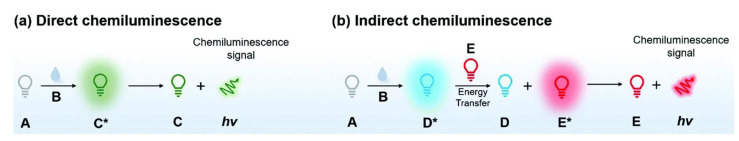
Schematic diagram of the mechanism of (**a**) direct and (**b**) indirect chemiluminescence. A: chemiluminescent molecule; B: response substance; C*: excited state of chemiluminescent species; C: ground state of the chemiluminescent species; D*: excited state of intermediate; D: ground state of intermediate; E*: excited state of fluorophores or fluorescent molecules; E: ground state of fluorophores or fluorescent molecules [17]. Reprinted with permission from Ref [17] Copyright 2020 the Royal Society of Chemistry, London, UK.

Chemiluminescence is a phenomenon of light emission (luminescence) that occurs because of chemical reactions of certain substances. Chemiluminescent molecules such as reactants or intermediates are activated by oxidation to form oxidized high-energy intermediates, which decompose and dissipate energy in light emission or transfer energy to surrounding fluorescent molecules. They finally return to their ground state, producing the phenomenon of chemiluminescence [18]. Therefore, chemiluminescence can generally be divided into two types according to the energy dissipation pathway: direct and indirect chemiluminescence (Figure 3) [25]. During direct chemiluminescence, the chemiluminescent molecule is oxidized to produce an unstable high-energy intermediate, an excited state of the luminescent species. Then, it dissipates energy back to the ground state by emitting photons. Many common chemiluminescent molecules, such as luminol, acridine esters, and their derivatives, can be oxidized by hydrogen peroxide to cause direct chemiluminescence. In contrast, the excited intermediate does not produce photon emission as it returns to the ground state in indirect chemiluminescence. However, it requires transferring energy to a nearby fluorophore and subsequent chemiluminescence. Chemiluminescent substrates first generate high-energy intermediates through chemical reactions, followed by the excitation of nearby fluorophores or fluorescent molecules through chemiluminescence resonance energy transfer (CRET), which release energy in the form of luminescence and return to the ground state. Various indirect luminescent substrates, such as 1,2-dioxetane derivatives and oxalate esters, are widely used to construct chemiluminescent systems combined with fluorescent systems for biomarker imaging or cancer therapy. 

## 5. CL-PDT System

The concept of the CL-PDT system was proposed to address the limitation of light penetration depth in PDT [21]. Like indirect chemiluminescence, chemiluminescent substrates and PSs are integrated into one system using covalent modification or noncovalent assembly (so-called CL-PDT system). When the chemiluminescent substrates are activated by oxidation, they excite the surrounding photosensitizer through the CRET process, generating ROS and realizing PDT without an external light source. CRET was first proposed in 1967 to simulate chemiluminescence as a fluorescent donor in Förster resonance energy transfer (FRET). It follows the rule of FRET, which states that the energy transfer efficiency is inversely proportional to the sixth power of the donor–acceptor distance [26,27]. Therefore, CRET has great potential for analyte detection, immunoassays, enzyme activity assays, in vitro and in vivo imaging, and disease diagnosis [28,29]. To construct a CL-PDT system, two conditions must be satisfied: (1) the distance between the chemiluminescent molecule (donor) and PS (acceptor) is 1–10 nm, and (2) the luminescence spectrum of the chemiluminescent molecule and the absorption spectrum of the PS should overlap; the larger the overlap region, the higher the probability of CRET. Currently, the strategies for establishing CL-PDT systems can be divided into covalent synthesis and noncovalent self-assembly. The available CL-PDT systems are summarized in Table 1.

### 5.1. Covalent CL-PDT System

The chemiluminescent molecule and appropriate PS are attached directly by chemical bonds for the covalent CL-PDT system. This form of CL-PDT system possesses good stability and a definite PDT effect. However, this system has some problems: (1) the synthesis is relatively complex, especially Schaap’s adamantylidene-dioxetane molecules; (2) the system lacks flexibility (the molecules need to be resynthesized when the application scenario changes); (3) the products may be toxic after the photochemical reaction.

Luminol can be covalently linked to PS in a single-molecule CL-PDT system owing to its low price and excellent derivatization properties. In the system, energy is directly transferred from luminol to PS. Akkaya’s group designed a luminol derivative with covalently modified erythrosine as a PS [30]. In the presence of hydrogen peroxide and copper ions, luminol was excited to transfer energy to erythrosine to produce ^1^O_2_. Unfortunately, the molecule was not extended to cells and animals. Zhang’s group synthesized an amphiphilic polymer (CLP) composed of luminol (chemiluminescent donor), chlorin e6 (a PS doubling as a fluorescent molecule), and polyethylene glycol [31]. The CLP self-assembled to form nanoparticles that exhibited excellent in vivo imaging capabilities in several animal models of inflammation and killed cancer cells by in situ ^1^O_2_ generated in tumor microenvironments with high levels of ROS. In addition, Zhang’s group applied CLP in vivo to luminescence imaging of tumors with high expression of hydrogen peroxide and triggered PDT in situ [32]. Algi’s group developed a heavy-atom-free chemiluminogenic PS composed of boron-dipyrromethene (BODIPY, a PS) and 2,3-dihydrophthalazine-1,4-dione (a luminol analog) to promote ^1^O_2_ production [33]. However, their molecules required an external light source to apply in vitro PDT. Luminol usually requires a catalyst such as metal ions, heme, or peroxidase in an alkaline solution to accelerate the reaction between luminol and hydrogen peroxide to enhance chemiluminescence intensity [46]. Luminol reacts only with ROS such as hydrogen peroxide and superoxide anion for instantaneous chemiluminescence. Thus, the CL-PDT system based on luminol and its derivatives is greatly restricted [47]. 

**Figure 4:**
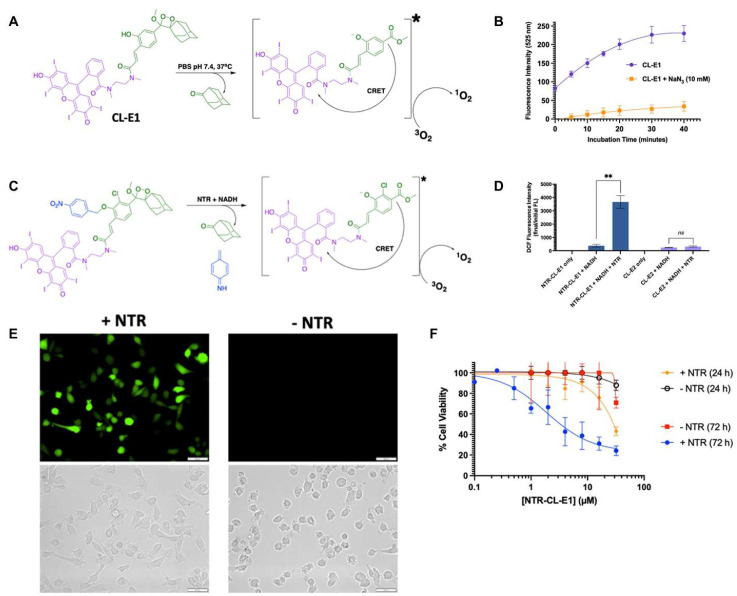
(**A**) Schematic illustration of ^1^O_2_ generation principle by CL-E1. (**B**) The ROS generation of CL-E1 by monitoring the fluorescence of 2′,7′-dichlorofluorescin in the absence (purple line) or presence (orange line) of sodium azide (a specific scavenger of ^1^O_2_). (**C**) Schematic illustration of ^1^O_2_ generation principle by NTR-CL-E1. (**D**) NTR-CL-E1 (blue) production of ROS (blue) after overnight incubation in the presence or absence of NTR. **: *p* < 0.01; ns: no significance. (**E**) ROS imaging of NTR-CL-E1 in MDA-MB231 cells +/− NTR expression (green fluorescence from 2′,7′-dichlorofluorescin). scale bar = 50 μm. (**F**) Cell viability of NTR-CL-E1 versus MDA-MB231 cells (+/− NTR-expressing) after 24 and 72 h of incubation. Reprinted with permission from Ref [34] Copyright 2022 American Chemical Society, New York, NY, USA.

Schaap’s adamantylidene-dioxetane is the only known chemiluminescent molecule with a stable dioxetane fraction, thus eliminating the need for an oxidation step [48,49,50]. Unlike other chemiluminescent molecules, Schaap’s adamantylidene-dioxetane can be equipped with protective groups that respond under specific conditions for different application requirements [51,52]. The efforts of researchers, particularly Doron Shabat, Kanyi Pu, Alexander Lippert, and Weihong Zhu, have developed various Schaap’s adamantylidene-dioxetane molecules with response conditions including ROS, reactive sulfides and nitrides, enzymes, gases, ions, and pH [17,18,53,54,55,56,57,58]. Regarding integrating Schaap’s adamantylidene-dioxetane into the PDT system, Beharry’s group contributed pioneering work and introduced the concept of ‘dark dynamic therapy’ [34]. They selected erythrosin B as a PS to anchor to Schaap’s adamantylidene-dioxetane (CL-E1). The phenolic hydroxyl group of Schaap’s molecule was deprotonated at pH 7.4, inducing erythrosine B to generate ^1^O_2_ via the CRET pathway (Figure 4A). CL-E1 was confirmed to produce ^1^O_2_, using 2′,7′-dichlorofluorescin as an ROS probe and sodium azide as a specific scavenger of ^1^O_2_ (Figure 4B). The experimentally obtained ^1^O_2_ efficiency of CL-E1 was 3.6 ± 0.51% (theoretical efficiency: 0.95%). MTT assays demonstrated that CRET-induced ^1^O_2_ production by CL-E1 could kill cancer cells (IC50 = 14 ± 2 μM for MCF7 cells). Further, they designed CL-PDT molecules (NTR-CL-E1) based on the CL-E1 core for nitroreductase (NTR) activation to produce ^1^O_2_ in specific tumor cell lines (Figure 4C). The NTR-CL-E1 could generate ROS in MDA-MB231 triple-negative breast cancer cells expressing NTR and inducing cancer cell death (IC50 = 1.9 ± 0.7 μM) based on the NTR activity (Figure 4D–F). Beharry’s work demonstrated the potential of Schaap’s molecules coupled to response groups for PDT, which is a milestone in expanding the covalent CL-PDT system.

### 5.2. Noncovalent CL-PDT System

Noncovalent chemiluminescent systems are prepared by supramolecular self-assembly of chemiluminescent molecules, PSs, and other constituent materials (often amphiphilic molecules). This strategy allows for a flexible mix of chemiluminescent molecules and PSs, and/or the incorporation of certain functional molecules as needed to achieve maximum PDT effects. The major advantage of noncovalent CL-PDT systems is the direct selection of commercially or clinically approved molecules, significantly reducing the risk of toxicity and facilitating clinical translation. However, concerns about the whole system’s stability in vivo and the energy transfer effectiveness between the chemiluminescent molecule and the PS are undeniable.

For noncovalent CL-PDT systems, the first luminol application as an energy donor for PDT was reported by Firer’s group [35]. They first constructed a biocouple consisting of transferrin and hematoporphyrin (Tf–Hp), which was significantly more specific for leukemia cells by a factor of nearly 7. Since the chemiluminescence spectrum of luminol overlaps well with the absorption spectrum of Tf–Hp, luminol was expected to activate PDT. Furthermore, luminol was found to have noticeable intracellular chemiluminescence, which lasted for 50 min. The combination of luminol (10 μM) and Tf–Hp conjugate (3 μM) was 95% cytotoxic against erythroleukemia cell lines. As the first noncovalent CL-PDT system, it is impressive that luminol application produces such superior PDT effects at the cellular level. However, there is an important issue to be clarified here: the pathway through which luminol transfers energy to hematoporphyrin. In any way, it is a pioneering work. Subsequently, different forms of PSs in combination with luminol have been obtained for various noncovalent CL-PDT systems, including 5-aminolevulinic acid [36], meta-tetra(hydroxyphenyl)-chlorin (encapsulated in folic acid and horseradish peroxidase-poly[2-methoxy-5-((2-ethylhexyl)oxy)-*p*-phenylenevinylene] dots) [37], poly[2-methoxy-5-(2-ethylhexyloxy)-1,4-phenylenevinylene] (encapsulated in hemoglobin-poly(styrene-*co*-maleicanhydride) fusogenic liposomes) [38], poly(lactic-co-glycolic acid) (co-precipitated with horseradish peroxidase, DSPE-mPEG2000 to format nanoparticles) [59], and Fe(III) Deuteroporphyrin IX chloride (co-assembled with polystyrene and cRGD-polystyrene-PEG) [39]. 

**Figure 5:**
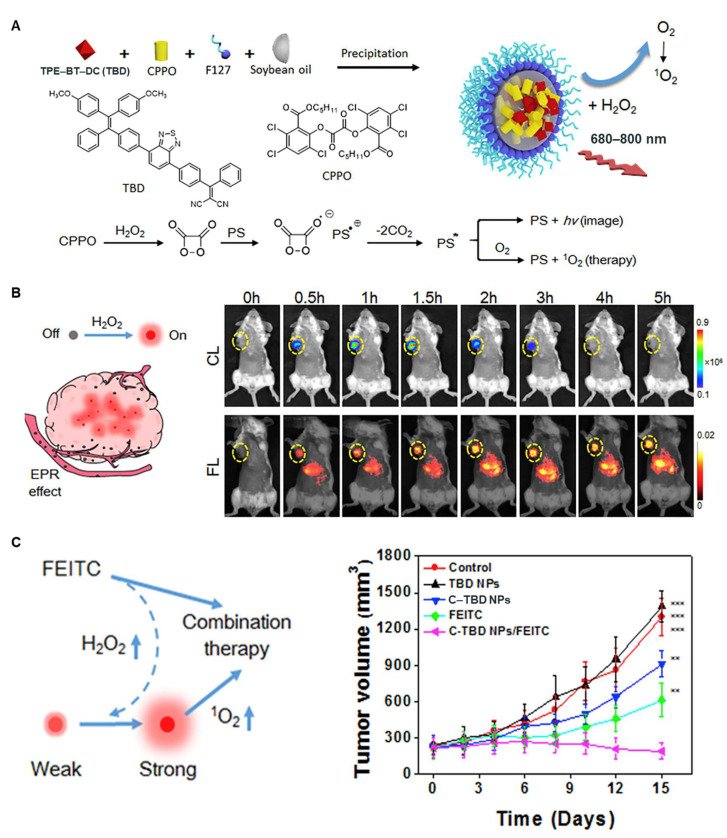
(**A**) Illustration of the preparation of C–TBD NPs and NIR imaging principle and PDT in the presence of hydrogen peroxide. (**B**) The tumor images of chemiluminescence and fluorescence. (**C**) The antitumor effects of C-TBD NPs in coordination with FEITC [43]. Reprinted with permission from Ref [43] Copyright 2017 Elsevier, Amsterdam, The Netherlands.

In addition to luminol, peroxyacetate derivatives have also been applied to build noncovalent CL-PDT systems [40,41,42,43,44,45]. Jiang’s group developed poly(ethyleneglycol)-poly(caprolactone) micelles to encapsulate peroxalate ester oligomer and mesotetraphenylporphine to bring them closer together, thereby transferring energy from peroxalate ester oligomer to mesotetraphenylporphine in the presence of hydrogen peroxide to produce ROS [45]. Similarly, Lee’s group co-assembled peroxalate polymer and protoporphyrin with pluronic F-127 to kill cancer cells [44]. Precision imaging and treatment are highly critical in the fight against cancer. The high level of hydrogen peroxide microenvironment within solid tumors affords the chemiluminescence system favorable selectivity for tumor imaging compared to normal tissue [17,60,61]. Liu’s group reported a nanomaterial (C-TBD NPs) with chemiluminescence-excited NIR emission and single-linear-state oxygen generation for precise theranostics of tumors [43]. As shown in Figure 5, the tetraphenylethylene derivative (TBD) was selected as a PS; bis[2,4,5-trichloro-6-(pentyloxycarbonyl)phenyl] oxalate (CPPO) was used as a chemical energy donor; soybean oil was used as a retardant to slow down the reaction rate between CPPO and hydrogen peroxide; and pluronic F-127 was used as a matrix to encapsulate the above components to form C-TBD NPs. CPPO was decomposed by hydrogen peroxide to form a high-energy 1,2-dioxetane intermediate, which excited TBD to produce NIR luminescence via a chemically initiated electron exchange luminescence process. In addition, the excited TBD could undergo intersystem crossing from its lowest singly excited state (S1) to the lowest triplet excited state (T1) and react with oxygen to form ^1^O_2_. In vivo studies demonstrated that C-TBD NPs could accurately image primary and metastatic tumors and exhibited significant antitumor effects in combination with β-phenylethyl isothiocyanate (FEITC).

## 6. Conclusions and Perspectives

The original goal of PDT was a noninvasive treatment modality with a promising future in treating malignant diseases such as tumors. In addition to being a primary treatment, PDT can also be used as an adjuvant therapy before or after surgery, chemotherapy, and radiotherapy to enhance the anti-cancer effects of conventional therapies. For specific diseases, such as certain skin diseases, the noninvasive nature of PDT will not only achieve or exceed the results of other treatment modalities but will also reduce the risk of skin damage and disfigurement. However, PDT is limited by light penetration depth and appears inadequate for diseases of deep tissues and internal organs. In response to the above, minimally invasive or more invasive techniques have been introduced in PDT, such as inserting optical fibers through surgical incisions. Of course, it is theoretically possible to achieve whole-body PDT with the approach, and there is no longer a question of light penetration depth as long as the patient can tolerate it. Nevertheless, such a crude means of solution abandons the original purpose of PDT design: noninvasive, minimal side effects, and easy operation. Therefore, developing PDT with unrestricted optical penetration depth is of profound value.

The chemiluminescence integrated with the PDT system may be an optimal solution. The related studies in our review tried to address the limitation of light penetration depth in PDT by activating PSs through chemiluminescence as “an internal light source”. As a proof of concept, researchers have confirmed that the CL-PDT system exhibits significant photodynamic effects in vitro and in vivo. Furthermore, these studies showed that introducing chemiluminescence into PDT avoids external light source limitations. 

However, the CL-PDT system is currently in its infant stage, and many critical issues must be addressed. Among them, the CL-PDT system loses one of the most significant advantages of PDT itself, namely high spatial and temporal resolution and light dose tunability. PDT uses an external light source that can selectively irradiate the lesion and adjust the irradiation time at any period according to the treatment effect. Moreover, the light source power can be regulated to ensure treatment effectiveness while reducing other toxic side effects. Therefore, it is a great challenge to retain the advantages of traditional PDT in the chemiluminescence-PDT system. For the above situation, the CL-PDT system needs to be optimized in two aspects: targeting ability and controlled activation. For targeting ability, preparation of nanoparticles or modification of targeting moieties enhances the selectivity of CL-PDT-like PSs and improves the drug concentration at the target site. CL-PDT can learn from this aspect of the technology, as it is well established in other areas. Regarding controlled activation, CL-PDT-like PSs based on Schaap’s adamantylidene-dioxetane molecules seem to be more promising. Schaap’s adamantylidene-dioxetane can be equipped with protective groups that respond to biomarkers. Therefore, introducing Schaap’s adamantylidene-dioxetane into PSs promises to solve the problem of selective activation of PDT. Notably, Doron Shabat’s group recently developed a new efficient synthetic route based on the Stille cross-coupling reaction for the preparation of adamantane chemiluminescents, which will greatly reduce the synthetic difficulty and expand the chemiluminescent molecular library [62].

Furthermore, it is crucial to improve the efficiency of the CL-PDT system. The process of CL-PDT can be roughly divided into four stages: chemiluminescent molecule activation (oxidation or protective group removal), energy transfer to PS, triplet PS generation, and ROS production. The coupling of all these steps will determine the overall yield of PDT. For example, the chemiluminescence quantum yield of Schaap’s dioxetane is only 0.003% in water [49]. In addition, the CRET-dependent CL-PDT system is sensitive to the distance between the acceptor and donor, which severely affects the energy transfer. In contrast, the study of PSs is relatively well established. Therefore, a better understanding of these issues is crucial for optimizing each step of current CL-PDT systems and developing highly efficient PDT. 

Finally, researchers should be aware of the chemiluminescent molecules’ toxicity and their reaction products. Luminol may cause damage to biological components such as DNA [63,64] and a high affinity for proteins [46,65,66,67]. Bis(2,4,5-trichloro-6-(pentyloxycarbonyl)phenyl)oxalate, bis(2,4,6-trichlorophenyl) oxalate, and bis-(2,4-dinitrophenyl) oxalate generate toxic products (phenolic compounds) to humans after chemiluminescence. In contrast, Schaap’s adamantylidene-dioxetane molecules have only been proven to be biocompatible in some cells and mice. These are open issues that need the attention of chemists and biomedical experts.

In summary, CL-PDT-related research is still at the proof-of-concept stage. The biggest advantage of the CL-PDT system is being free of an external light source, compared to conventional PDT. Researchers should attempt to apply the CL-PDT system more to the treatment of deep-seated diseases in vivo in future investigations. For instance, various tissues or organs where light cannot reach are the most suitable application scenarios for CL-PDT. In addition to the functions of PDT, CL-PDT also has the ability to image diseases with high contrast because of the unique advantages of chemiluminescence itself. In addition to PDT, the CL-PDT system is also capable of imaging disease sites with high contrast capabilities during treatment, owing to the unique advantages of chemiluminescence itself. Therefore, innovations in basic chemiluminescence theory, development of new chemiluminescent molecules, optimization of energy transfer, targeted enrichment and controlled activation, and biocompatibility must be studied and addressed in future work. 

## Figures and Tables

**Table 1:** Available CL-PDT systems.

Type	Chemiluminescent Substrate	PS	Cell	IC50 μM	Animal	Disease	Ref.
Covalent CL-PDT	luminol derivative	erythrosine	–	–	–	–	[30]
	luminol	chlorin e6	RAW264.7 B16F10 MOVAS MCF-7 A549	498.8 414.2 267.9 153.0 123.7	male BALB/c mice C57BL/6 mice BALB/c nude mice	ulcerative colitis tumor	[31]
	luminol	chlorin e6	4T1 HCT116 A549	53.8 67.3 124.0	Male BALB/c mice	tumor	[32]
	luminol derivative	BODIPY	Hep-2	–	–	–	[33]
	Schaap’s adamantylidene-dioxetane	erythrosin B	MCF7 MDA-MB231	14.0 1.9	–	–	[34]
Noncovalent CL-PDT	luminol	hematoporphyrin	Friend’s Leukemia K-562 U-7.6	0.08 0.3 0.5	–	–	[35]
	luminol	5-aminolevulinic acid	Caco-2	–	–	–	[36]
	luminol	meta-tetra(hydroxyphenyl)- chlorin	MCF-7, C6, NIH 3T3	–	–	–	[37]
	luminol	poly [2-methoxy-5-(2- ethylhexyloxy)-1,4-phenylenevinylene]	Hela	–	–	–	[38]
	luminol	Fe(III) Deuteroporphyrin IX	HeLa, MCF-7, H1299 cells, RAW264.7	–	nude mice	tumor	[39]
	TCPO	chlorin e6	HT29 cells	–	male BALB/c nude mice	tumor	[40]
	CPPO	DPAC-S	293T, KYSE-150	–	–	–	[41]
	CPPO	tetraphenylporphyrin	HeLa	–	BALB/c nude mice	tumor	[42]
	CPPO	TBD	4T1	–	BALB/c mice	tumor	[43]
	HPOX	protoporphyrin	SW620	–	–	–	[44]
	Peroxalate ester oligomer	mesotetraphenylporphine	LoVo	–	–	–	[45]

TCPO: bis [3,4,6-trichloro2-(pentyloxycarbonyl)phenyl]oxalate; CPPO: bis [2,4,5-trichloro-6-(pentyloxycarbonyl)phenyl] oxalate; DPAC-S: 4,4’-(dibenzo[a,c]phenazine-9,14-diyl) pyridin-1-ium bromide; TBD: TPE-BT-DC; HPOX: hydroxybenzyl alcohol-incorporating copolyoxalate.
